# Cardiac output‐guided maternal position therapy for preterm labor—it’s time for a trial

**DOI:** 10.1111/aogs.14365

**Published:** 2022-05-06

**Authors:** Thomas L. Archer

**Affiliations:** ^1^ San Antonio TX USA

**FIGURE 1:**
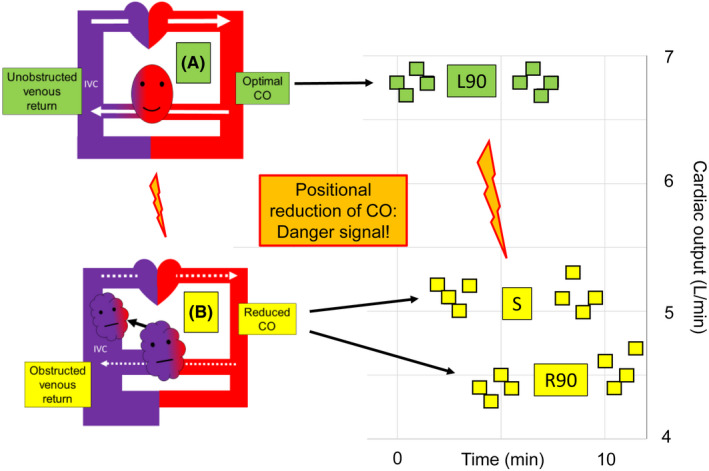
Positional and reproducible maternal cardiac output reductions can serve as a warning signal for positional obstruction of the inferior vena cava by the gravid uterus Note: The positional maternal cardiac output (CO) data obtained by electrical cardiometry and shown in this Figure have been simplified, approximated and re‐sequenced for didactic purposes, but are based on real data from the case report of a 34‐year‐old patient admitted to the hospital because of preterm labor at 26+3/7 weeks of gestation.^2^ Her weight was 84 kg, height 170 cm and body mass index 29. She was not having contractions during the CO measurements, was comfortable in all positions and had received no anesthesia or other medications. Additionally, the fetal heart rate tracing was normal in all maternal positions. As suggested in this Figure, the original positional CO changes were reproducible, but they occurred in another sequence than that shown here. In this Figure the patient spends 2 min lying in full left lateral decubitus position (L90), in which her CO is maximal because her inferior vena cava (IVC) is unobstructed. She then turns supine (S) for 2 min, during which her uterus partially obstructs the IVC, causing an increase in uterine venous pressure and a decrease in both CO and uterine perfusion. Two minutes later, when the patient turns into the full right lateral decubitus position (R90), her CO decreases even more and her uterine perfusion may deteriorate even further. The patient then turns back into full left lateral decubitus position and repeats the cycle of maximal uterine perfusion followed by impaired uterine perfusion. It may be that such cycles of relative uterine ischemia followed by reperfusion may trigger and maintain preterm labor, as well as exacerbating other pathologies of pregnancy such as intrauterine growth restriction, stillbirth, placental abruption, dysfunctional labor and uterine infection or unfavorable changes in the uterine microbiome. CO, cardiac output; IVC, inferior vena cava; L90, patient lying on her left side; R90, patient lying on her right side; S, patient supine. Oval smiling face (A) represents well oxygenated myometrium with low venous pressure and swollen unhappy face (B) represents edematous, congested and hypoxic myometrium

*Sir*,

In a previous letter, I proposed that prevention of positional inferior vena cava (IVC) obstruction by the gravid uterus during gestation might prevent or alleviate a variety of pregnancy‐related disorders.^1^ Obstruction of the IVC by the uterus—in the absence of adequate lumbar and azygos venous collaterals—can cause four interrelated harmful conditions: (1) increased uterine venous pressure, (2) decreased venous return to the heart, (3) decreased cardiac output (CO) and (4) decreased uterine arterial pressure. The dangers of this pathophysiological complex have been understood since the 1950s, but detection and correction of IVC obstruction has been difficult and empirical. Trending maternal CO using emerging non‐invasive technology may be a valuable tool in obstetrics, since a reproducible positional reduction in maternal CO can serve as an objective warning signal for IVC obstruction and thereby counsel maternal avoidance of that position. Figure 1 visually summarizes our conception of how the trending of CO might be used to detect occult obstruction of the IVC by the uterus, together with images of the state of the myometrium, without and with obstructed perfusion.^2^, ^3^

But how could obstruction of the IVC by the gravid uterus cause preterm labor? Obstruction of the IVC causes uterine venous congestion and hypoxia, and hypoxia—in general—suppresses myometrial contractility. Resolution of this apparent paradox might be provided by the description of "hypoxia‐induced force increase" during normal labor, in which cycles of myometrial ischemia and reperfusion cause progressive strengthening of contractions.^4^ The triggering and maintenance of preterm labor might be a variation on this theme, as the gravid uterus alternately blocks and allows abundant myometrial perfusion, depending on patient position.

Therefore, I propose a study of position therapy for preterm labor, comparing gestational age at birth for two groups of hospitalized patients, randomized to receive either standard care or standard care plus cardiac output‐guided maternal positioning. In the cardiac output‐guided group, the patient would be positioned to maintain not only an optimal fetal heart rate tracing but also maternal CO at its maximum baseline level. Of course it is possible that once preterm labor has been triggered it is too late to be stopped by position therapy, and this possibility might justify attempts to prevent the onset of preterm labor in the first place by performing positional maternal CO studies in patients at high risk, such as the morbidly obese.

Four findings are consistent with this ischemia and reperfusion model for preterm labor. First, activation of inflammatory pathways in the uterus is known to precede normal labor and ischemia and reperfusion do cause inflammation; secondly, maternal obesity is associated with IVC obstruction, inflammation and preterm labor; thirdly, premature birth is more common in first than in subsequent pregnancies; and fourthly, aerobic exercise appears to decrease preterm birth in obese women.^5^

Cardiac output‐guided positioning might be inconvenient for the patient, but the knowledge that avoiding certain positions favors her baby’s long term wellbeing would make it more acceptable.
